# A Long Non-coding RNA Lnc712 Regulates Breast Cancer Cell Proliferation

**DOI:** 10.7150/ijbs.36429

**Published:** 2020-01-01

**Authors:** Yue Cui, Chunxiao Lu, Zhiming Zhang, Aiqin Mao, Lei Feng, Li Fu, Feng Gu, Xin Ma, Dongxu He

**Affiliations:** 1 Wuxi School of Medicine, Jiangnan University, Wuxi, China.; 2 School of Food Science and Technology, Jiangnan University, Wuxi, China.; 3 Department of Breast Cancer Pathology and Research Laboratory, State Key Laboratory of Breast Cancer Research, Cancer Institute and Hospital, Tianjin Medical University, Tianjin, China.

**Keywords:** Long non-coding RNAs, Breast Cancer, HSP90, CDK2

## Abstract

Great quantity of intergenic noncoding RNAs (lncRNAs) have been identified in the mammalian genome and involved in various biological processes, especially in the development and metastasis of cancer. In this study, we identified one lncRNA, lncRNA NONHSAT028712 (Lnc712), was highly expressed in breast cancer cell lines and tissues based on microarray screening. Knockdown of Lnc712 largely inhibited breast cancer cell proliferation. Mechanistically, Lnc712 bound specifically to heat-shock protein 90 (HSP90). Interaction between Lnc712 and HSP90 is required for HSP90 binding to cell division cycle 37 (Cdc37). The Lnc712/HSP90/Cdc37 complex regulated cyclin-dependent kinase 2 (CDK2) activation and then triggered breast cancer cell proliferation. In summary, our results identified a new lncRNA regulate breast cancer proliferation though interaction with HSP90.

## Introduction

Breast cancer is one of the most common malignant tumors among women 1; it is the fifth leading cause of cancer-related deaths worldwide, with an increasing incidence annually 2. Breast cancer is a heterogeneous carcinoma, and both genetic and epigenetic changes contribute to its initiation and development. However, the underlying molecular and biological functions correlated with breast cancer are still unclear. Consequently, finding therapeutic targets is a major challenge.

Studies suggest that protein-coding genes account for <2% of the human genome, whereas the vast majority of transcripts are non-coding RNAs 3, 4. Long non-coding RNAs (lncRNAs), ranging from 200 nucleotides to 100 kb, constitute the largest proportion of ncRNAs. LncRNAs regulate the transcription process by interacting with RNA-binding proteins, co-activating transcription factors, and repressing the promoters of target genes 5-7. It has been suggested that lncRNAs play key roles in tumorigenesis 8.

In this study, we identified the novel function of one lncRNA, NONHSAT028712 (Lnc712), and showed that it plays a role in breast cancer cell proliferation. Up-regulation of Lnc712 was involved in breast cancer proliferation *via* the HSP90-Cdc37-CDK2 pathway.

## Materials and methods

### Microarray

The microarray profiling was conducted in the laboratory of OE Biotechnology Co. (Shanghai, China). RNA was separately extracted from MCF-7/ADM and MCF-7 cells using the acid-phenol and chloroform method. Cyanine-3-CTP-labeled cRNA was obtained using a Quick Amp labeling kit (Agilent Technologies, Santa Clara, CA, USA) and then purified with an RNeasy Mini kit (Qiagen, Valencia, CA, USA). The labeled cRNAs were then hybridized onto Agilent-062918 OE Human lncRNA Microarray V4.0 028004 (Agilent Technologies), which is a Custom Gene Expression Array for OE Biotechnology Co. and detects 46,506 lncRNAs. After washing, the arrays were scanned with an Agilent scanner (G2505C).

### Cells and Cultures

Human breast epithelial cells (MCF-10A; American Type Culture Collection (ATCC)), the human breast cancer cells MDA-MB-231 and MCF-7 (ATCC), and long-term adriamycin 9-treated MCF-7 cells (MCF-7/ADM) were cultured in Dulbecco's modified Eagle's medium (Gibco, Gaithersburg, MD, USA) supplemented with 10% fetal bovine serum (Sijiqing, Hangzhou, China), 100 μg/mL penicillin, and 100 U/mL streptomycin (Beyotime, Shanghai, China) at 37℃ in a humidified atmosphere with 5% CO_2_. MCF-7/ADM cells were generated by treating MCF-7 cells with stepwise increasing concentrations of ADM over 8 months.

### RNA extraction and RT-qPCR analysis

Total RNA was extracted with TRIzol according to the manufacturer's instructions (Invitrogen, Carlsbad, CA, USA). RNA was precipitated with GlycoBlue (AM9516, Ambion, Waltham, MA, USA) and dissolved in diethylpyrocarbonate-treated water. Samples of 1 μg of DNase-treated RNA were reverse-transcribed using a PrimeScript RT reagent kit (RR047A, Takara, Shiga, Japan). qPCR was performed using SYBR Green mix (RR890A, Takara) with the cycling conditions 95℃ for 30s followed by 40 cycles of 95℃ for 5 s and 60℃ for 30 s.

LncNONHSAT028712: forward, 5΄-AAATACCTCACCCTCATCTATACCAAC-3΄; reverse, 5΄-TTTCCCGTTGCCATTGAT-3΄.

CDK2 (cyclin-dependent kinase 2): forward, 5΄-CGCTTGTTAGGGTCGTAGTG-3΄; reverse, 5΄-AGATTGACCAGCTCTTCCGG-3΄.

GAPDH: forward, 5'-CAAGAAGGTGGTGAAGCAGG-3΄; reverse, 5΄-TCAAAGGTGGAGGAGTGGGT-3΄.

### Patients and specimens

For fluorescence *in situ* hybridization (FISH) validation of Lnc712, 10 fresh breast cancer and paired non-cancer tissue samples were collected at the Tianjin Tumor Hospital from 2017 to 2018. Inclusion criteria were patients with primary breast cancer, having tumor stage I-IV, and surgery was the initial treatment approach. Informed consent for the use of samples was given by all patients, and the study was approved by the Ethics Committee of the Institute of Basic Medical Sciences, Chinese Academy of Medical Sciences.

### Fluorescence *in situ* hybridization

FISH was performed using the lncRNA FISH Probe Mix kit (Ribobio, Guangzhou, China). Briefly, sections were deparaffinized, dehydrated in 100% ethanol, and dried. Slides were incubated with hydrogen peroxide for 30 min at room temperature (RT), then subjected to protease digestion for 20 min and dehydrated in an ethanol series. The prehybridization buffer was applied to a selected area on each slide, and incubated at 37°C for 2 h. The slides were incubated with hybridization buffer for co-denaturation of lncRNA and probe RNA at 37°C for 16 h. After hybridization, the slides were washed with saline-sodium citrate buffer and then mounted in anti-fade solution with DAPI.

### RNA-protein pull-down assays

Biotinylated lncRNAs were refolded in structure buffer [10 mM Tris·HCl, pH 7.0, 10 mM MgCl_2_, 0.1 M KCl]. The diluted RNAs were incubated at 95℃ for 2 min, put on ice for 3 min, and left at RT for 30 min. For pull-down incubation, lysates containing 1 mg protein were pre-cleared with streptavidin beads and then incubated with 2 μg biotinylated RNA and streptavidin beads for 1 h at RT. The beads were collected by centrifugation and washed three times with buffer Ⅱ [50 mM Tris·HCl, pH 7.0, 1 mM EDTA, 100 mM KCl, 0.1% TritonX-100, 5% glycerol, 1 mM DTT]. RNA-associated proteins were eluted and resolved on SDS/PAGE followed by silver staining according to the manufacturer's instructions (Bio-Rad Laboratories, Hercules, CA).

### Reverse Pull-Down Assays

Lysates of MCF-7/ADM cells were prepared as described for RNA pull-down assays^10^. Samples containing 1 mg protein were precleared with protein A/G beads for 1 h at 4°C. HSP90 or mouse IgG-specific antibody (5 μg/sample) was mixed with protein A/G beads and incubated with cell lysate overnight at 4°C. The captured RNAs were purified using Protease K and phenol/chloroform precipitation; extracted RNAs were then subjected to Northern blot analysis.

### Small-interfering RNA transfection

Cells were transiently transfected with gene-specific or scrambled siRNA using Lipofectamine 2000 transfection reagent (ThermoFisher, Waltham, MA, USA) following the procedure recommended by the manufacturer as described previously 19. Lnc712 was silenced using an individual siRNA targeting the sequence 5′-GACCAGATATCCCTCGCTT-3′; HSP90 was silenced using an individual siRNA targeting the sequence 5′-GGAACGTGATAAAGAAGTA-3′.

### Cell vitality assays

Cells were plated into 96-well plates and grown overnight at 37℃ under 5% CO_2_. After transfection with siRNA for 48 h, 10 μl MTT (3-(4,5-dimethylthiazol-2-yl)-2,5-diphenyltetrazolium bromide) was added to each well and incubated for 4-6 h at 37℃. Then the medium was removed and 100 ml dimethyl sulfoxide was added to each well. The optical density (OD) was measured with a microplate reader (Bio-Rad Laboratories, Hercules, CA) at 570 nm to assess the relative number of surviving cells.

### Cell-cycle analysis

Cells were seeded in 6-well plates and cultured in medium at 37℃ under 5% CO_2_. Forty-eight hours after transfection, the cells were trypsinized and washed twice with cold phosphate-buffered saline (PBS). Then the cells were fixed in 1000μl 70% ethanol for 2 h at 4℃, followed by the addition of 500μl cell-cycle staining buffer (Beyotime, Nantong, China). After 30-min incubation at 37°C, a flow cytometer (BD Accuri^TM^ C6, Mountain View, CA) was used to analyze the DNA content of the cells.

### Western blot

Cells were lysed in RAPI (Sigma-Aldrich, St. Louis, MO, USA) containing 1% phenylmethylsulfony fluoride (Beyotime, Shanghai, China) and centrifuged for 15 min at 4℃. Protein concentrations were then measured using a BCA protein assay kit (Beyotime). Proteins were separated on 12% gels using sodium dodecyl sulfate polyacrylamide gel electrophoresis. For immunoblots, the PVDF membrane carrying the transferred proteins was incubated at 4℃ overnight with designated primary antibodies diluted in TBST buffer containing 0.1% Tween20 and 5% bovine serum albumen (BSA). Immunodetection was accomplished using a horseradish peroxidase-conjugated secondary antibody and an enhanced chemiluminescence detection system (P1108, Beyotime). The antibodies anti-CDK2 (ab32147), anti-HSP90 (ab13492), anti-Cdc37 (ab224831), and anti-ubiquitin (ab134953) were from Abcam, and anti-GAPDH (AP0063) was from Bioworld Technology Inc. (St. Louis Park, MN, USA). Images were analyzed using ImageJ. (National Institutes of Health, Bethesda, MD, USA).

### Co-immunoprecipitation

Forty-eight hours after transfection, cells were harvested and lysed in RIPA base buffer [50 mM Tris·HCl, pH 7.4, 137 mM NaCl, 0.25% sodium deoxy cholate, 4% protease inhibitor cocktail]. Antibodies were incubated overnight with cell lysates at 4°C on a rotating platform. Forty microliters of protein A/G plus-agarose magnetic beads (Santa Cruz Biotechnology) was incubated with cell lysates for 4 h at 4℃, then the beads were washed three times with PBS and the bound proteins were eluted by boiling for 5 min with 50 µl SDS sample buffer. Samples were then subjected to Western blot analysis.

### Animal experiments

MDA-MB-231 cells were injected subcutaneously into the flanks of female BALB/c mice (5 × 10^6^ cells/mouse). All mice were housed under air-filtered, pathogen-free conditions. Tumor volumes were estimated using the formula: volume (mm^3^) = 3.14 × (width)^2^ × length/6. When the tumors reached ∼200 mm^3^, the animals were also injected once every 3 days at the tumor sites with Lnc712 siRNA or scrambled siRNA (5 nmol) as control.

### Immunofluorescence analysis

After incubation with 5% BSA, the slides were incubated with primary antibody overnight at 4°C in a humidified chamber, followed by the appropriate secondary fluorescence-labeled antibody (Invitrogen) for 3 h at room temperature. Nuclei were counterstained with DAPI (Beyotime). Images were captured on a Zeiss confocal microscope.

### Statistical analyses

Results are presented as mean ± SD. We assessed comparisons between groups using one-way ANOVA or Student's t-test. We performed the statistical analyses using Graphpad Prism 5.0 software. All statistical tests were two-sided, and P values <0.05 were considered to be statistically significant.

## Results

### Lnc712 is up-regulated in breast cancer

**Figure 1:**
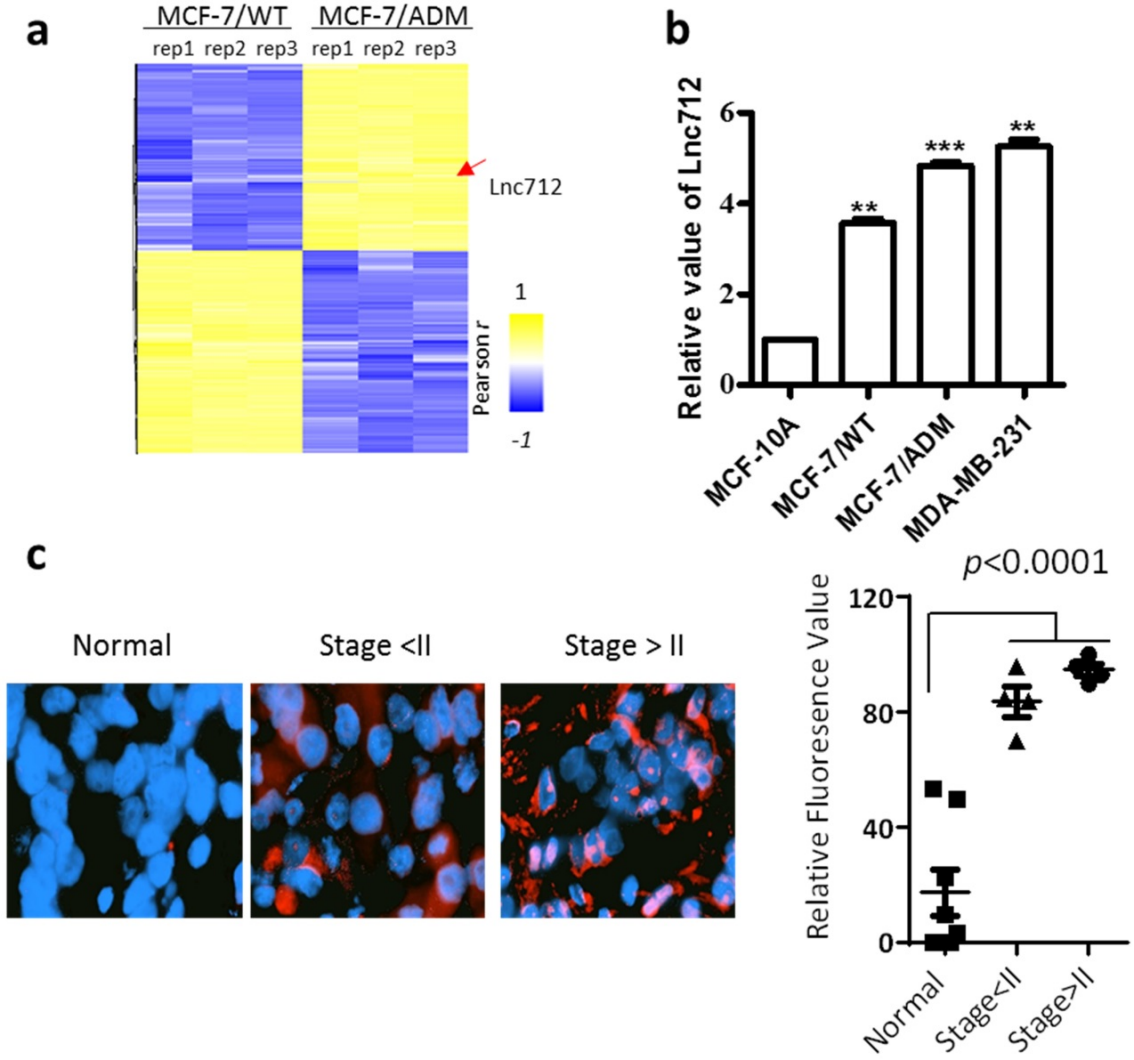
** Lnc712 was up-regulated in breast cancer.** (a) Hierarchical clustering of distinct lncRNAs in MCF-7/ADM and MCF-7/WT cells. Profiling was replicated 3 times. (b)RT-qPCR validated the expression of Lnc712 in normal breast epithelial cell and breast cancer cells, the values for MCF-10A cells were normalized to 1. Results are mean ± SD. **P < 0.01, ***P <0.001. (c) Lnc712 expression in breast cancer tissues and adjacent normal tissues were detected using FISH. n=10.

An lncRNA microarray was used to analyze the expression of lncRNAs in breast cancer cells. In order to identify lncRNAs involved in breast cancer cell proliferation, progression or chemoresistance development, we compared the lncRNA profiles from MCF-7 cells and MCF-7/ADM cells. MCF-7/ADM cells are generated by long-term treatment with adriamycin, and they have a greater ability to proliferate, metastasize and resistance to chemotherapeutic drugs than the parental control MCF-7 cells (MCF-7/WT) 11, 12. Therefore, LncRNAs differentially expressed in MCF-7/ADM cells plays as candidates that regulates cancer proliferation, progression or chemoresistance. Among the 60 most differentially-expressed lncRNAs, NONHSAT028712 (Lnc712) was significantly higher in MCF-7/ADM cells (Fig. 1a, also see raw microarray data in GSE81971), and the result was validated with RT-PCR (Fig. 1b). Interestingly, unlike other chemoresistance-related proteins (e.g. p-glycoprotein 13 and glutathione S -transferase P1 14) that expressed at a low level in chemosensitive cancer cells, RT-PCR results showed although Lnc712 was higher in MCF-7/ADM cells, it is also expressed in MCF-7/WT cells (Fig. 1b). Therefore, Lnc712 may participate general signaling pathways involved in cancer proliferation and progression. To validate such assumption, we analyzed Lnc712 expression in another breast cancer cells line, MDA-MB-231 cells, and then compared Lnc712 expression in different breast cancer cells with that in non-tumorigenic epithelial cell line MCF-10A. As expected, the Lnc712 expression was lowest MCF-10A cells, while it was significantly higher in all three breast cancer cell lines, and it was highest in the more malignant MCF-7/ADM and MDA-MB-231 cells. Together, these results suggest a role of Lnc712 in breast cancer development.

We then validated the Lnc712 expression in clinical samples (Supplementary Table 1). RNA-FISH demonstrated that Lnc712 expression was significantly higher in breast cancer tissue than in adjacent normal tissue (Fig. 1c). Together, these results indicated that Lnc712 might relate to breast cancer progression.

### Lnc712 is associated with heat-shock protein 90

**Figure 2:**
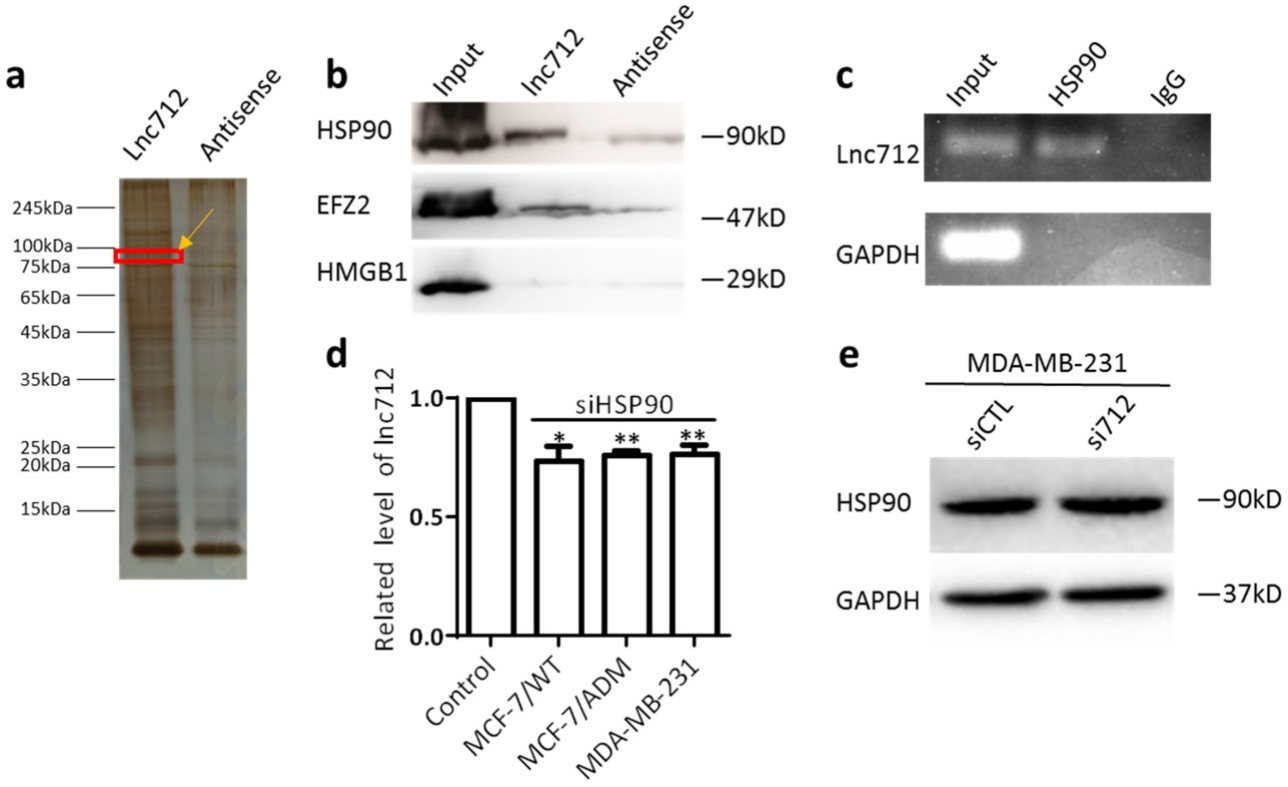
** Lnc712 is associated with the HSP90 protein.** (a)Silver staining of biotinylated Lnc712-associated proteins. (b)Western blotting of proteins from Lnc712 pull-down assays. (c) RIP experiments were performed using the HSP90 antibody to probe RNA from MDA-MB-231 cell extracts, and the levels of the coprecipitated RNAs were determined using qRT-PCR. (d )Lnc712 expression in HSP90 knockdown cells. Results are mean ± SD. **P<0.01. (e)Western blot analysis of the effect of Lnc712 knockdown on HSP90 in MDA-MB-231 cells.

RNA-FISH was then used to determine the localization of Lnc712, and it was found mainly in the cytoplasm (Supplementary Fig. 1), Suggesting this lncRNA may regulate cytosolic biological process, e.g. binding to cytosolic factors, rather than direct regulate gene expression. To understand the mechanism of action of Lnc712, we sought to identify intracellular Lnc712-binding factors using pull-down assays. Biotinylated Lnc712 (or a negative control RNA) was incubated with total protein extracts from MCF-7/ADM cells and pulled down with streptavidin beads to identify the associated proteins by silver staining (Fig. 2a). We chose four areas of bands for analysis by mass spectrometry, which later identified HSP90, EFZ2, and HMGB1 as Lnc712-binding proteins as confirmed by immunoblotting the proteins that were captured in the pull-down assay (Fig. 2b). Among these, HSP90 as a molecular chaperon that controls activation or ubiquitination of a client protein resulting in proteasomal degradation 15, were most specific. HSP90 is overexpressed in many cancers compared to its expression in normal tissues 16, 17. HSP90-specific antibody also was able to enrich the endogenous Lnc712 in extracts from MCF-7/ADM cells (Fig. 2c).

To further understand the interaction between the Lnc712-HSP90 comp lex, three types of breast cancer cell were transfected with Lnc712 siRNA or HSP90 siRNA and the knockdown efficiency was confirmed by RT-qPCR and Western blot (Supplementary Fig. 2a and b). Since Lnc712 has a highly homologous gene that expresses GST3 on chromosome 11, we further validated the specificity of siRNA (Supplementary Fig. 2c). Interestingly, we found that Lnc712 expression was decreased in cells with HSP90-knockdown (Fig. 2d), suggesting that HSP90 is required for Lnc712 function and stabilization. On the contrary, knockdown of Lnc712 did not affect HSP90 expression (Fig. 2e and Supplementary Fig. 2d). Together, these results indicated that Lnc712 and HSP90 form a functional ribonucleoprotein complex.

### Lnc712-HSP90 regulates breast cancer cell proliferation *via* the CDK2 pathway

**Figure 3:**
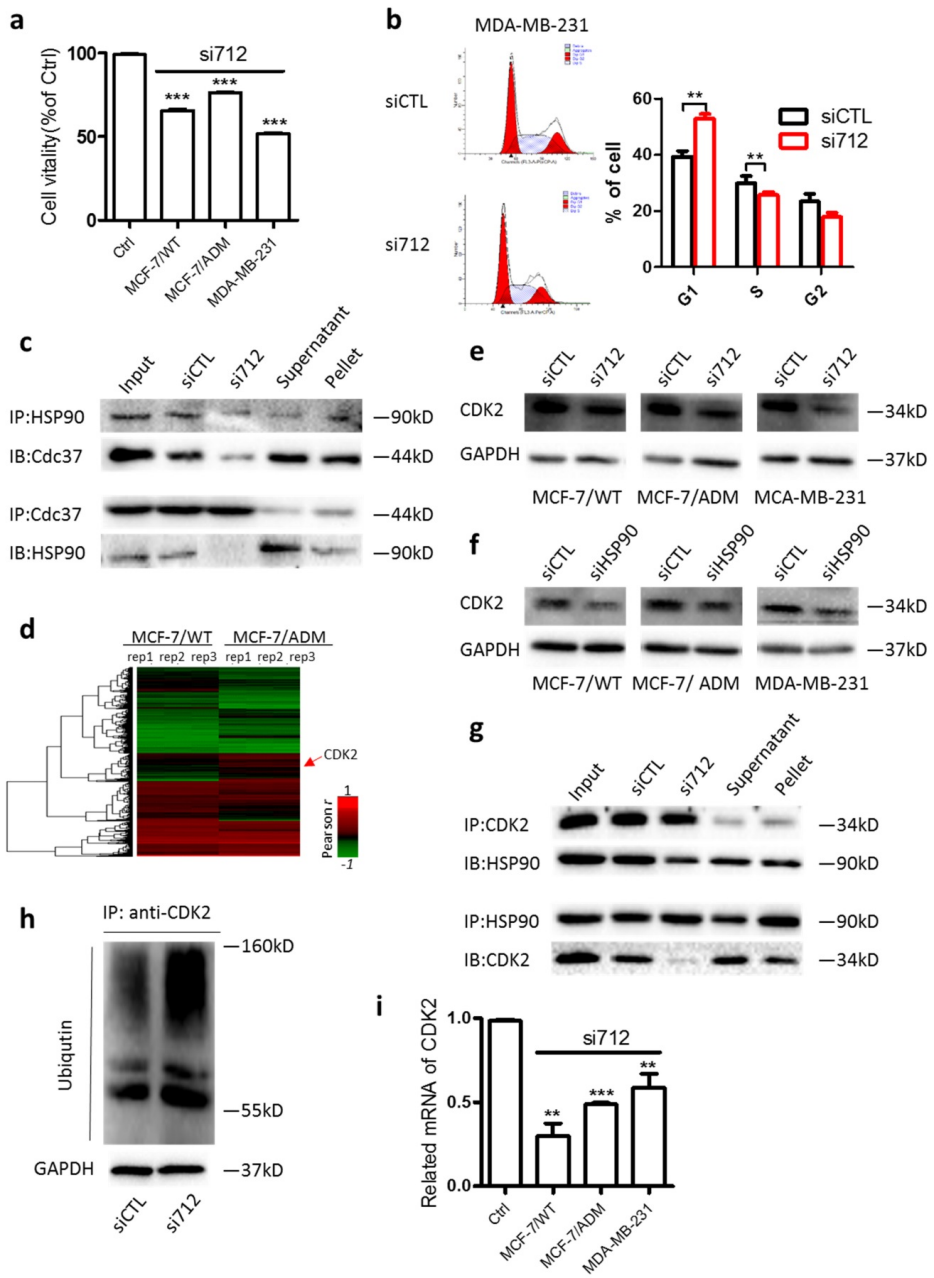
** Lnc712-HSP90 regulate breast cancer cell proliferation via CDK2 pathway.** (a) Cell vitality assays were used to determine the cell viability of Lnc712 siRNA transfected MCF-7, MCF-7/ADM and MDA-MB-231 cells. Results are mean ± SD of two independent experiments with duplicate wells. ***P <0.001. (b) Flow cytometry was performed to determine the effect of Lnc712 on changes in cell cycle distribution. (c)HSP90 and Cdc37 interaction was studied in MDA-MB-231 cells transfected with Lnc712 siRNA or scrambled siRNA by Co-IP. (d) Hierarchical clustering of distinct mRNAs in MCF-7/ADM and MCF-7/WT cells. Profiling was replicated 3 times. (e) Western blot for CDK2 in three breast cancer cells that transfected with Lnc712 siRNA. (f) Western blot for CDK2 in three breast cancer cells that transfected with HSP90 siRNA.(g) HSP90 and CDK2 interaction was studied in MDA-MB-231 cells transfected with Lnc712 siRNA or scrambled siRNA by Co-IP. (h) Knockdown of Lnc712 increases ubiquitination of CDK2 in MDA-MB-231 cells. (i) CDK2 mRNA level was decreased when cells were treated with Lnc712 siRNA.

HSP90 stabilizes a number of proteins required for tumor growth. Knockdown of HSP90 decreased the viability of MCF-7, MC-7/ADM, and MDA-MB-231 cells (Supplementary Fig. 3a). Similarly, we found that after Lnc712 expression was decreased by siRNA, the viability of MCF-7, MC-7/ADM, and MDA-MB-231 cells was markedly decreased (Fig. 3a). The decreased viability was due to cell-cycle arrest at G0/G1 (Fig. 3b and Supplementary Fig. 3b). These results suggest that Lnc712 is required for the function of HSP90 in regulating tumor growth.

Regarding the action of HSP90 in cell-cycle regulation, cell division cycle 37 (Cdc37) is one of the best-studied co-chaperones of HSP90 18-21. As expected, HSP90 co-immunoprecipitated with Cdc37 in MDA-MB-231 cells, while knockdown of Lnc712 significantly decreased the HSP90-Cdc37 interaction (Fig. 3c), suggesting that Lnc712 interacts with HSP90 and reinforces the stability of the HSP90-Cdc37 complex.

Nearly 300 client proteins of HSP90-Cdc37 has been identified. We then calculated the correlation between the expression of lncRNAs and coding genes using the database GSE81971. CDK2, a known client protein of HSP90-Cdc37 22, was then identified because its expression was strongly positively correlated with high level of Lnc712 (Fig. 3d). To validate, we found CDK2 protein level was higher in breast cancer cells than normal breast cells (Supplementary Fig. 3c). Importantly, the CDK2 protein level was decreased by treatment with either Lnc712 or HSP90 siRNA (Fig. 3e and f). The downregulation of CDK2 protein by Lnc712 might be attributable to ubiquitination of the protein as previous studies suggested that functional HSP90-CDC37 acts to stabilize activate CDK2 23. Indeed, we found in Coimmunoprecipitation assay that HSP90-CDK2 interactions was decreased after Lnc712 knockdown (Fig. 3g). Such changes induced by siLnc712 then abolished the stabilization of CDK2 by HSP90-CDC37, and CDK2 was ubiquitinated (Fig. 3h). The decreased CDK2 protein was accompanied by a reduced level of CDK2 mRNA (Fig. 3i). Together, these results indicate that Lnc712 interacts with HSP90 and controls the activity of CDK2.

**Figure 4:**
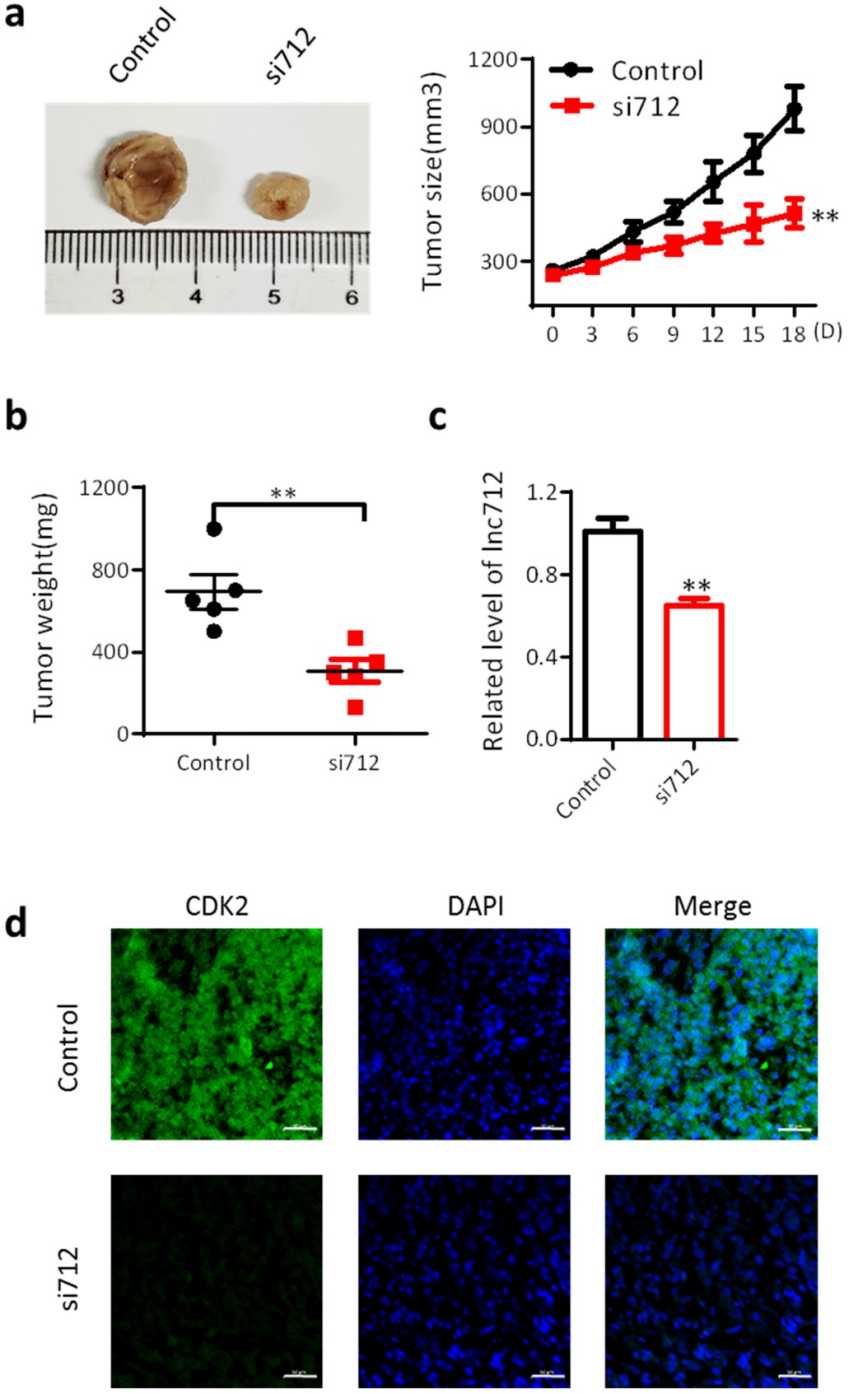
** Lnc712 promotes breast cancer cell growth *in vivo*.** (a) Representative figure and Growth curves for the xenograft of Lnc712-knockdown tumor. (b)Tumor weights of the xenograft Lnc712-knockdown tumors were measured. (c) RT-qPCR for Lnc712 level in MDA-MB-231 xenograft. (d) Immunofluorescence images showing the intensity of CDK2 expression in tumors from xenografted mice. scale bar, 50 µm.

Finally, we investigated the effect of Lnc712 on the proliferation of breast cancer cells *in vivo*. BALB/c mice were used as an *in vivo* model of mammary carcinoma. The mouse tumor model was generated by intradermal injection of MDA-MB-231 cells. Five mice were treated with Lnc712 siRNA twice a week for 3 consecutive weeks. After 3 weeks, tumors were collected for measurements of tumor size and weight. Tumors treated with Lnc712 siNRA were significantly smaller than control tumors (Fig. 4a and b). The knockdown effects of Lnc712 siRNA were confirmed by measuring the Lnc712 levels (Fig. 4c). Immunohistochemical analyses of randomly-selected xenograft MDA-MB-231 tumors from nude mice demonstrated that tumors with Lnc712 knockdown expressed lower levels of CDK2 (Fig. 4d). Thus, Lnc712 promotes breast cancer cell growth *in vivo*.

## Discussion

The complete sequencing of the human genome revealed many surprises, and increasing numbers of lncRNAs have been identified 24. Researches have demonstrated that lncRNAs participate in various cellular processes, including the regulation of epigenetic signatures, gene expression, and proliferation 25, 26. In breast cancer, many lncRNAs such as MIAT (myocardial infarction-associated transcript), SNHG1 (small nucleolar RNA host gene 1), and MALAT1 (metastasis-associated lung adenocarcinoma transcript 1) have been reported to participate in the occurrence, progression, and metastasis of tumors 27-29. This means that lncRNAs are emerging as potential biomarkers for the diagnosis, prognosis, and therapy of breast cancer.

**Figure 5:**
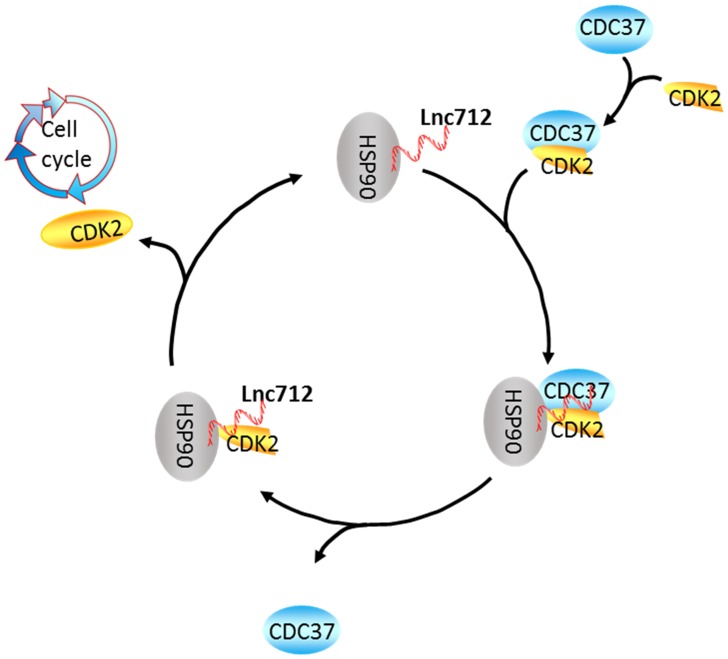
** A speculative model of the Lnc712-HSP90-Cdc37 cycle.** Cdc37 first establishes connection with the CDK2 to create a Cdc37-CDK2 complex. Then, the binary complex binds to Hsp90-Lnc712 to form a stable complex. The formation of the Hsp90-Cdc37-CDK2 complex finally facilitates CDK2 maturation. Lnc712 helps HSP90 to participate in the interaction with Cdc37 and CDK2.

At the beginning of the experiments, we tried to identify chemoresistance-related lncRNAs, and then identified a higher expression of Lnc712 in chemoresistance MCF-7/ADM cells than in chemosensitive MCF-7/WT cells. By seeking the related mechanism, we found Lnc712 directly control the proliferation of MCF-7/ADM cells, making them more vulnerable to the stress of adriamycin. Because 'high proliferative' is universal to all cancer cells regardless their chemo-response, so we then tried to explore the role of Lnc712 in regulating breast cancer cell proliferation and progression. We found that Lnc712, located on chromosome 12, is highly expressed in breast cancer, then investigated its oncogenic role by evaluating its effects on the proliferation of breast cancer cells. We demonstrated that Lnc712 knockdown inhibited proliferation and growth through cell-cycle-arrest in G0/G1. Moreover, we also found that CDK2 protein levels were positively correlated with Lnc712, indicating that Lnc712 regulates the cell cycle by regulating CDK2. CDK2 is critically involved in cell cycling and has been proposed as a potential cancer target 30. Thus, Lnc712 may play a key role in regulating the development and progression of breast cancer (Fig. 5).

It is well-known that HSP90 is one of the major molecular chaperones in eukaryotic cells 31. It is an important component of the chaperone machinery, which facilitates protein folding and regulates the stabilization and activity of numerous client proteins 32-34. HSP90 client proteins originate from distinct functional classes, including transcription factors, kinases, and steroid hormone receptors. Many of these client proteins are commonly overexpressed in cancer cells 35. Many studies have shown that the formation of lncRNA-protein complexes is an important process by which lncRNAs carry out their functions. An RNA-protein complex can assemble chromatin-modifying complexes 36 and modulate mRNA stability 37, 38. Based on our results, Lnc712 functions by forming an RNA-protein complex with HSP90.

Co-chaperones are key regulators that drive the diverse functions of the HSP90 chaperone machinery. These co-chaperones, including HOP, Cdc37, SGTA, TAH1, and p23, in concert with HSP90 form complexes that direct a broad range of specific clients to HSP90 32. Cdc37 is the best-studied of these co-chaperones, and is considered to be exclusively associated with protein kinases 39. Recent progress has allowed the interactions of HSP90-Cdc37-client protein to be determined 19, 40. To date, nearly 300 client proteins of HSP90-Cdc37 have been found, and CDK2 is one of them 22. Our results show that Lnc712 reinforces the interactions of HSP90 and Cdc37 to regulate CDK2 expression.

In recent years, HSP90 has become a potential target for a number of diseases including cancer, neurodegeneration, and infectious diseases 41-44. The development of HSP90 inhibitors has grown rapidly. For example, 17-DMAG binds to HSP90 and inhibits its function, which eventually results in the degradation of HSP90 client proteins 45; KU675, a novel C-terminal HSP90 inhibitor, has potent anti-proliferative and cytotoxic activity along with client protein degradation, without the induction of a heat-shock response in both androgen-dependent and -independent prostate cancer cell lines 46; and triptolide inhibits HSP90β in a three-fold manner to decrease the CDK4 protein levels in HeLa cells, causing a reduction in the phosphorylation of Rb, and resulting in cell-cycle-arrest at G1 47. So far, none of the inhibitors of HSP90 have been approved for clinical application in cancer, because systematic inhibition of HSP90 activity may lead to high cytotoxicity mainly due to dysfunction in heat-shock responses 48, 49. However, our research may provide new strategy for inhibiting cancerous HSP90 by targeting Lnc712, which is specifically highly expressed in breast cancer cells but not in surrounding healthy tissues. In future works, it is worth investigating the expression of Lnc712 in different breast cancer subtypes, and also in other types of cancers to acquire better understanding of Lnc712 as an anti-cancer target.

Our results reveal a novel therapeutic target for human breast cancer. Destabilizing the HSP90-Cdc37 complex to arrest the cell-cycle by reducing the binding of Lnc712 to HSP90 is a new approach.

## Supplementary Material

Supplementary figures and tables.
