# A Long Non-coding RNA Lnc712 Regulates Breast Cancer Cell Proliferation: Erratum

**DOI:** 10.7150/ijbs.81665

**Published:** 2023-01-05

**Authors:** Yue Cui, Chunxiao Lu, Zhiming Zhang, Aiqin Mao, Lei Feng, Li Fu, Feng Gu, Xin Ma, Dongxu He

**Affiliations:** 1 Wuxi School of Medicine, Jiangnan University, Wuxi, China.; 2 School of Food Science and Technology, Jiangnan University, Wuxi, China.; 3 Department of Breast Cancer Pathology and Research Laboratory, State Key Laboratory of Breast Cancer Research, Cancer Institute and Hospital, Tianjin Medical University, Tianjin, China.

After the publication of our article, we detected one unintentional error during the preparation of Fig. S2B in PowerPoint. Western blot bands of GAPDH in MCF-7/WT were run on a same gel with that of MCF-7/ADM, but the images were mistakenly inserted. After carefully checked the original data, a correction was made. This error did not change the data or conclusions of the article in any way.

Furthermore, Prof. F Gu was the major supervisor of clinical experiments designing and performing, with the permission of Prof. L Fu. However, we are deeply grieved by the passing of Prof. F Gu. In this situation, Prof. L Fu personally feels she cannot give full advices during the process of corrigendum or in future without detailed experimental information from Prof. F Gu. Therefore, all authors agreed to remove authorship of Prof L Fu and F Gu.

We apologize for the inconvenience for these changes.

## Figures and Tables

**Figure 1:**
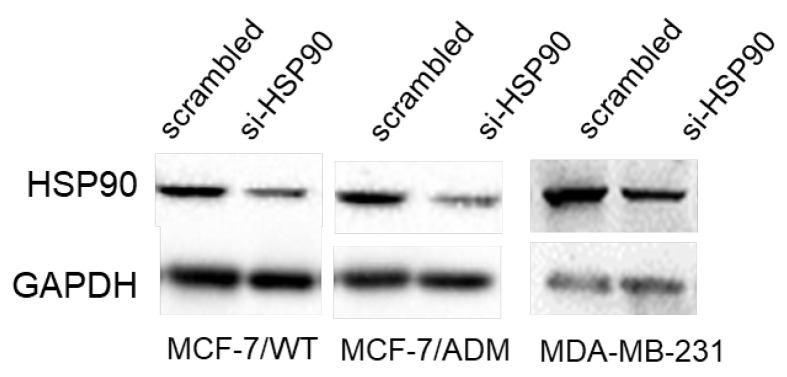
Correct Fig. S2B. Western blotting analysis the expression of HSP90 in HSP90 siRNA transfected three breast cancer cells.

