# Supplementary material for: A Long Non-coding RNA Lnc712 Regulates Breast Cancer Cell Proliferation
Source: Int J Biol Sci. 2020 Jan 1;16(1):162–71. doi: 10.7150/ijbs.36429 (PMC6930380; doi:10.7150/ijbs.36429)
Supplement: Supplementary file 1 — Supplementary figures and tables. [file ijbsv16p0162s1.pdf]

s1

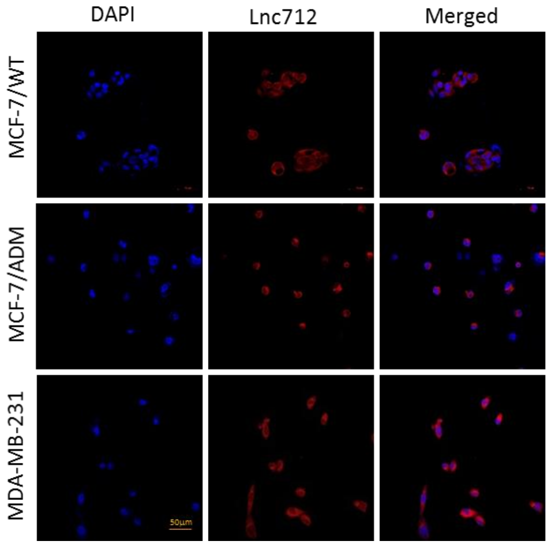

Fig.s1 Fluorescence in situ hybridization of cells performed using a Lnc712 probe (red). The nuclei were counterstained using DAPI(blue). scale bar, 50 μm.

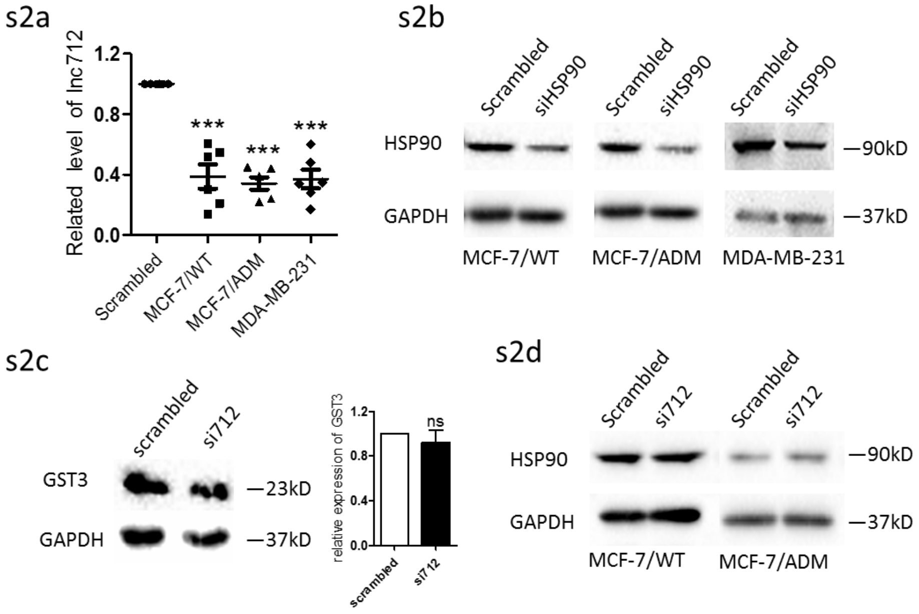

Fig.s2a RT-qPCR analysis the expression of Lnc712 in Lnc712 siRNA transfected three breast cancer cells. n=3. Fig.s2b Western blotting analysis the expression of HSP90 in HSP90 siRNA transfected three breast cancer cells. Fig.s2c Western blotting analysis the expression of GST3 in Lnc712 siRNA transfected breast cancer cells. Fig.s2d Western blot analysis of the effect of Lnc712 knockdown on HSP90 in MCF-7 cells.

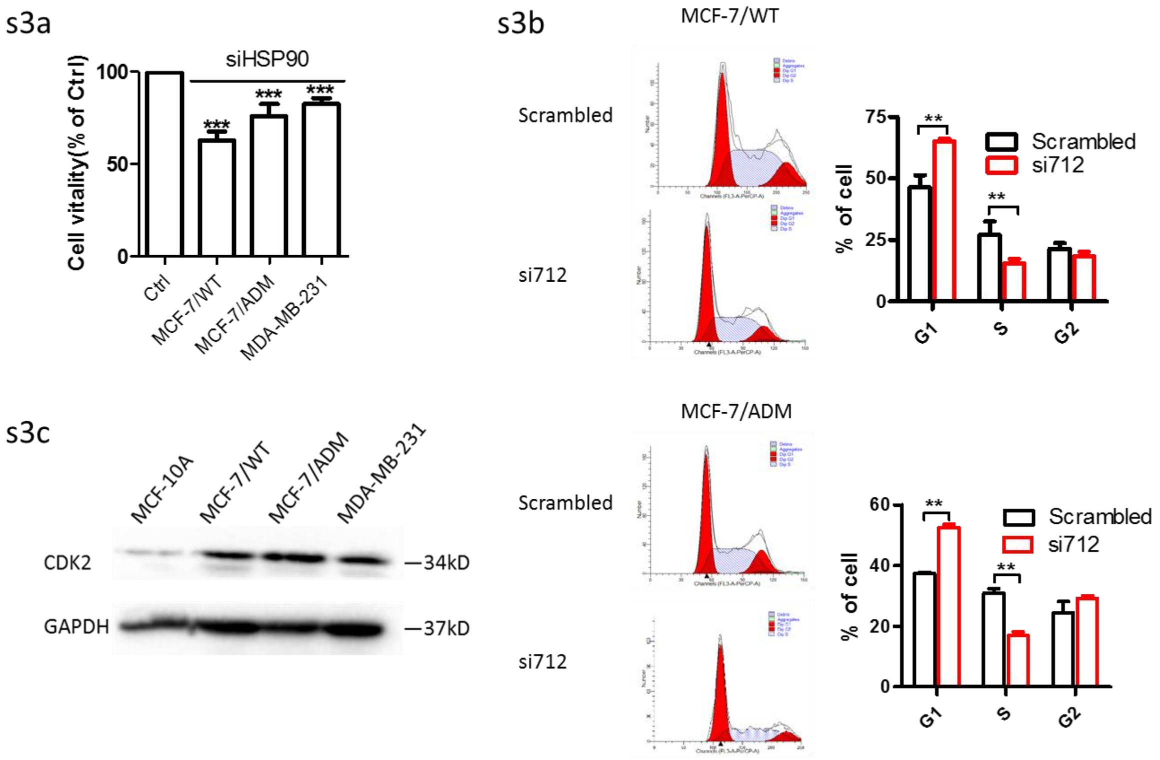

Fig.s3a Cell vitality assays were used to determine the cell viability of HSP90 siRNA transfected three breast cancer cells. n=3 Fig.s3b Flow cytometry was performed to determine the effect of Lnc712 on changes in cell cycle distribution. Fig.s3c Western blot show that CDK2 expression in breast cancer cells is higher than normal epithelial cells.

Table S1 Information for clinical samples

	All Patients (n=10)
Age (years)	
<50	6
≥50	4
Sex	
Male	0
Female	10
T stage	
T1	6
T2	4
Lymph node status	
N0	4
N1	5
N2	0
N3	1
AJCC Substage	
I	0
II	8
III	2

Tab.s1 Tumor characteristics for breast cancer patients.
